# Validation of Breast Cancer Risk Models by Race/Ethnicity, Family History and Molecular Subtypes

**DOI:** 10.3390/cancers14010045

**Published:** 2021-12-23

**Authors:** Anne Marie McCarthy, Yi Liu, Sarah Ehsan, Zoe Guan, Jane Liang, Theodore Huang, Kevin Hughes, Alan Semine, Despina Kontos, Emily Conant, Constance Lehman, Katrina Armstrong, Danielle Braun, Giovanni Parmigiani, Jinbo Chen

**Affiliations:** 1 Department of Biostatistics, Epidemiology & Informatics, University of Pennsylvania, Philadelphia, PA 19104, USA; yi.liu@emory.edu (Y.L.); sarah.ehsan@pennmedicine.upenn.edu (S.E.); jinboche@pennmedicine.upenn.edu (J.C.); 2 Department of Biostatistics, Harvard T. H. Chan School of Public Health, Harvard University, Boston, MA 02115, USA; guanz@mskcc.org (Z.G.); jwliang@g.harvard.edu (J.L.); thuang@ds.dfci.harvard.edu (T.H.); dbraun@mail.harvard.edu (D.B.); gp@jimmy.harvard.edu (G.P.); 3 Department of Data Sciences, Dana-Farber Cancer Institute, Boston, MA 02215, USA; 4 Massachusetts General Hospital, Boston, MA 02114, USA; hughkevi@musc.edu; 5 Newton Wellesley Hospital, Newton, MA 02462, USA; ASEMINE@PARTNERS.ORG (A.S.); clehman@partners.org (C.L.); karmstrong6@mgh.harvard.edu (K.A.); 6 Department of Radiology, University of Pennsylvania, Philadelphia, PA 19104, USA; Despina.Kontos@pennmedicine.upenn.edu (D.K.); Emily.Conant@pennmedicine.upenn.edu (E.C.)

**Keywords:** breast cancer, risk prediction, mammography

## Abstract

**Simple Summary:**

Several statistical models exist to predict a person’s risk of breast cancer. Risk assessment models can guide cancer screening approaches by identifying individuals who would benefit from additional screening. In this study, we compared the performance of four models in predicting the 5-year risk of breast cancer in a cohort of women aged 40–84 years who underwent screening mammography at three large health systems. Models showed comparable discrimination (ability to distinguish between cases and non-cases) and calibration (ability to accurately predict risk) overall, with no difference by race. Model discrimination was poorer for some cancer subtypes, and better for women with high BMI. The combined BRCAPRO+BCRAT model had improved calibration and discrimination among women with a family history of breast cancer. Our results can inform risk-based screening approaches by identifying women at a high risk of breast cancer.

**Abstract:**

(1) Background: The purpose of this study is to compare the performance of four breast cancer risk prediction models by race, molecular subtype, family history of breast cancer, age, and BMI. (2) Methods: Using a cohort of women aged 40–84 without prior history of breast cancer who underwent screening mammography from 2006 to 2015, we generated breast cancer risk estimates using the Breast Cancer Risk Assessment tool (BCRAT), BRCAPRO, Breast Cancer Surveillance Consortium (BCSC) and combined BRCAPRO+BCRAT models. Model calibration and discrimination were compared using observed-to-expected ratios (O/E) and the area under the receiver operator curve (AUC) among patients with at least five years of follow-up. (3) Results: We observed comparable discrimination and calibration across models. There was no significant difference in model performance between Black and White women. Model discrimination was poorer for HER2+ and triple-negative subtypes compared with ER/PR+HER2−. The BRCAPRO+BCRAT model displayed improved calibration and discrimination compared to BRCAPRO among women with a family history of breast cancer. Across models, discriminatory accuracy was greater among obese than non-obese women. When defining high risk as a 5-year risk of 1.67% or greater, models demonstrated discordance in 2.9% to 19.7% of patients. (4) Conclusions: Our results can inform the implementation of risk assessment and risk-based screening among women undergoing screening mammography.

## 1. Introduction

While breast cancer mortality has fallen over the past decade in the U.S., it remains the second leading cause of cancer death among women, with 43,600 breast cancer deaths projected in 2021 [1]. Identification of patients at high risk of developing breast cancer could allow targeting of preventive and screening interventions to mitigate risk to further reduce mortality. Specifically, risk-based screening approaches that tailor screening initiation, interval, and supplemental screening to individual risk may increase benefits and reduce harms of screening. Multiple validated risk assessment models have been developed to quantify an individual woman’s risk of developing breast cancer [2]. The Breast Cancer Risk Assessment Tool (BCRAT, also known as the Gail model) utilizes age, race/ethnicity, history of breast biopsy and atypical hyperplasia, first-degree family history of breast cancer, age at menarche, and age at first birth to estimate risk [3,4,5,6]. The Breast Cancer Surveillance Consortium (BCSC) model [7] utilizes age, race/ethnicity, first-degree family history of breast cancer, breast biopsy and benign breast disease, and breast density, as measured by the American College of Radiology Breast Imaging Reporting and Database System [8]. The BRCAPRO model [9] was developed in the setting of women undergoing genetic counseling and uses a detailed family history of breast and other cancers to estimate risk both of a *BRCA1/2* mutation and risk of breast cancer. Recently, the BRCAPRO model was combined with the BCRAT model to create the BRCAPRO+BCRAT model [10] that incorporates both factors in the BCRAT model and detailed family history.

Our prior work compared the BCRAT, BCSC, and BRCAPRO models in a cohort of women undergoing mammography screening at a single institution [11]. We found comparable moderate discrimination and good calibration of these three models; however, we were unable to assess differences in model performance by race/ethnicity due to the lack of diversity in the cohort. Few studies have evaluated the performance of breast cancer risk models by race/ethnicity. Additionally, while we found poorer predictive accuracy of the models for HER2+ and triple-negative breast cancers, the numbers of cancers of these subtypes were small, limiting our ability to draw strong conclusions.

The purpose of the current study is to compare the performance of breast cancer risk models for use in the setting of mammography screening, and specifically to evaluate model performance among subgroups defined by race/ethnicity, by molecular subtypes, by family history, and by obesity. Additionally, we performed validation of the recently developed BRCAPRO+BCRAT model [10], utilizing three large mammography screening cohorts. We evaluated the performance of BCRAT, BCSC, BRCAPRO, and BRCAPRO+BCRAT models because these models utilize risk factors that are routinely collected during the course of clinical care in these three health systems, and therefore the integration of one or more of these risk models into clinical care for decision making is potentially feasible.

## 2. Materials and Methods

### 2.1. Study Population

We assembled three cohorts of women presenting for screening mammography at Massachusetts General Hospital (MGH), Newton-Wellesley Hospital (NWH), and the University of Pennsylvania Health System (UPenn) between 2006–2015, 2006–2015, and 2011–2015, respectively. All mammograms included were digital or digital breast tomosynthesis... The following patient-reported risk factors were collected via questionnaire at the time of the mammogram: age, race/ethnicity, age at menarche, age at first birth, body mass index (BMI), history of breast biopsy, history of atypical hyperplasia or benign breast findings, and family history of breast cancer. Breast density measurements were pulled from radiology reports in electronic medical records (EMR) and were classified based on the American College of Radiology’s Breast Imaging-Reporting and Data System (BI-RADS). EMR was also used to supplement missing survey information on the history of breast biopsy, atypical hyperplasia, BMI, and history of prior breast cancer. At MGH, the questionnaire asked whether women had ever had “benign tissue removed from the breast” which was considered as evidence of atypical hyperplasia or benign breast findings. Additionally, at MGH and NWH, we additionally included data from pathology reports on diagnoses of atypical hyperplasia, lobular carcinoma in situ (LCIS), or lobular neoplasia. At UPenn, women were asked whether they had previously been diagnosed with atypical hyperplasia. Additional detail about benign breast conditions was not available for UPenn. Missing BMI specifically was supplemented with the closest measurement from EMR within 1 year prior to or 6 months after the mammogram. The presence of BRCA1 and BRCA2 pathogenic genetic mutations was also collected from linkage with genetic counseling records. We included the first mammogram visit over the time period for each woman, using covariates collected at the time of the first mammogram visit in the cohort for analysis (N = 91,094 at MGH; N = 54,032 at NWH; N = 48,035 at UPenn).

**Figure 1:**
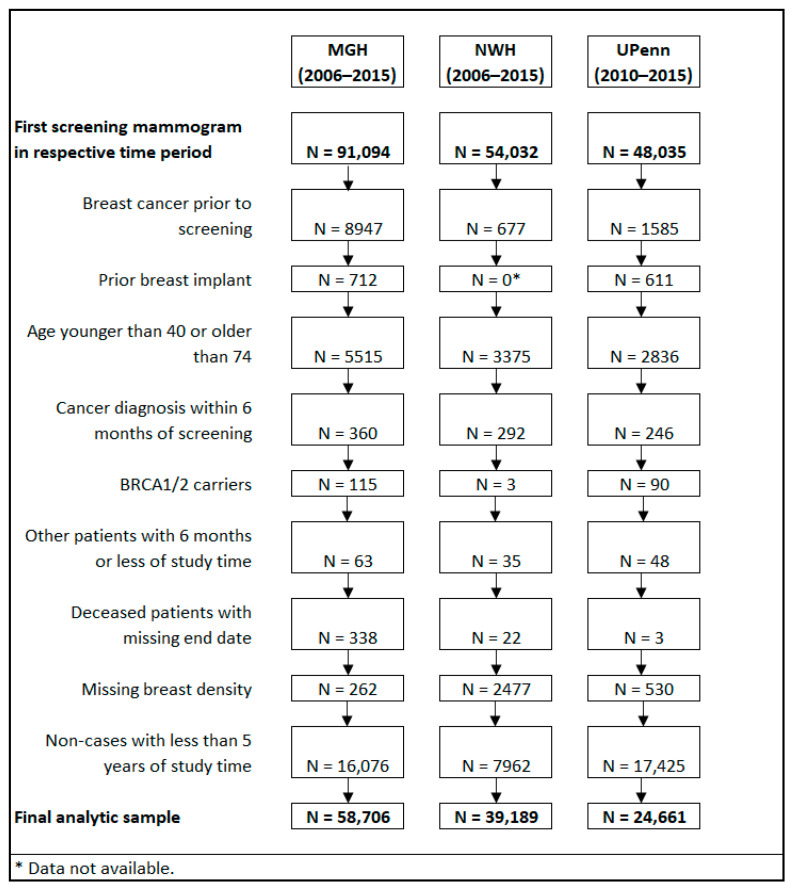
Exclusion criteria for screening mammography population by study site.

Patients with breast cancer prior to screening were excluded, as were patients diagnosed with cancer within 6 months of screening (Figure 1). For MGH and UPenn, patients with breast implants were excluded; information on breast implants was not available for NWH. Patients who did not fall between the ages of 40 and 84 were excluded, as were those with known pathogenic BRCA1 or BRCA2 mutations since the BCRAT and BCSC models are not appropriate for women with these mutations. Patients with less than 5 years of follow-up time and deceased patients with no date of death or no date of the last contact were also excluded. Patients with missing BI-RADS breast density were excluded. These exclusions resulted in final analytic samples of 58,706 patients from MGH, 39,189 patients from NWH, and 24,661 patients from UPenn (Figure 1).

### 2.2. Outcomes

Breast cancer cases were determined through to 31 December 2017, using a combination of hospital cancer registries and state health department cancer registries. At MGH and NWH, breast cancer diagnosis information was obtained from the Massachusetts Cancer Registry, while at UPenn, breast cancer diagnosis information was obtained from Pennsylvania, New Jersey, and Delaware state cancer registries. Invasive breast cancers were categorized into molecular subtypes based on the expression of estrogen receptor (ER), progesterone receptor (PR), and human epidermal growth factor receptor 2 (HER2). Borderline ER and PR results were considered positive, while borderline HER2 results were considered unknown [12]. Since this analysis predicted a 5-year absolute risk of breast cancer, only patients with invasive breast cancer within 5 years were considered cases. Patients with ductal carcinoma in situ (DCIS) were not considered as cases, since the risk models considered the risk of invasive breast cancer.

### 2.3. Statistical Analysis

All patients were followed from the date of first screening mammogram visit until breast cancer diagnosis, death, or administrative censoring on 31 December 2017. We estimated the 5-year absolute risk of breast cancer using the BCRAT, BCSC, BRCAPRO, and BRCAPRO+BCRAT models and compared the results with observed breast cancer outcomes. Death was treated as a competing risk. We used the BCRA R package (v2.1) for BCRAT (https://dceg.cancer.gov/tools/risk-assessment/bcra, accessed on 23 November 2021); the BayesMendel R package (v2.1-7) for BRCAPRO and BRCAPRO+BCRAT (https://projects.iq.harvard.edu/bayesmendel/bayesmendel-r-package, accessed on 23 November 2021); and the BCSC SAS program (v2.0) for BCSC (https://tools.bcsc-scc.org/BC5yearRisk/sourcecode.htm, accessed on 23 November 2021).

We assumed patients with missing data on atypical hyperplasia had no atypical hyperplasia (N = 59,285, 48.37%) and that those with a missing number of biopsy examinations had no such examinations (N = 18,549, 15.22%). Regarding missing menopause status, any person over 55 or anyone who had stopped menstruating was categorized as postmenopausal [13]. Anyone who did not meet this criterion was categorized as premenopausal. For patients without, or with incorrect, or ambiguous family history information (approximately 3% total), we assumed that they had no affected relatives. 

We validated and compared the performance of BCRAT, BCSC, BRCAPRO, and BRCAPRO+BCRAT with respect to the absolute risk of invasive breast cancer. We also compared these models in subgroups defined by race/ethnicity, invasive molecular subtypes (ER/PR+HER2−, ER/PR+HER2+ or ER/PR−HER2+, or ER/PR/HER2−), a number of affected first- or second-degree relatives, age <50 or ≥50 years, and BMI <30 kg/m^2^ or ≥30 kg/m^2^. We performed sensitivity analyses evaluating model performance by site and comparing performance for Black and White women only at UPenn since the majority of Black women were from that site.

Calibration of risk prediction models was evaluated by the observed to the expected ratio (O/E), where the numerator is the observed count of invasive breast cancer cases that arose within 5 years of the initial mammogram, and the denominator is the expected number of cases predicted by the model, obtained by the sum of the predicted 5-year absolute risk estimates. An O/E ratio of 1 indicates perfect calibration, an O/E ratio >1 indicates that the model under-predicts the true number of cases, and an O/E ratio <1 indicates that the model over-predicts the true number of cases. Calibration curves were plotted as the observed proportion of breast cancer cases versus the predicted proportion of breast cancer cases in each decile of predicted absolute risk. Discrimination was assessed by area under the receiver operating characteristic curve (AUC). AUC measures the probability that a prediction model provides higher absolute risk scores for cases than for non-cases. An AUC of 0.5 indicates that the model performs no better than chance, while an AUC of 1 indicates perfect discrimination. The clinically recognized threshold for elevated 5-year risk of breast cancer for chemoprevention is 1.67% [14,15]. This cutoff was used in calculating the true positive rate (TPR) (sensitivity) and the false positive rate (FPR (1— specificity)). TPR was calculated as the proportion of cases who were categorized as high-risk by the model and the FPR was calculated as the proportion of non-cases who were categorized as high-risk by the model.

To obtain 95% confidence intervals (CI) for the performance metrics we generated bootstrap samples and calculated metrics for each model in each bootstrapped sample [16]. To test the statistical significance of observed differences in AUCs across models and strata, we calculated the difference in AUC between two models or two strata for each bootstrap replicate, then calculated the test statistic as the observed difference in AUCs squared, divided by the variance of the difference in AUC derived from the bootstrapped samples. Using this statistic, we obtained *p*-values for the difference in AUC between pairwise comparisons of models and strata using the Chi-square distribution with 1 degree of freedom. All statistical tests were two-sided using an alpha of 0.05. In addition, we adjusted for multiple comparisons (we performed a total of 34 model and strata comparisons) by the Bonferroni method, with corrected *p*-values less than 0.00147 considered statistically significant (*p* = 0.05/34 comparisons).

We also compared the proportion of patients categorized as high vs. low absolute risk, comparing two models at a time and using the cutoff of 1.67% 5 years risk. All analyses were performed using R statistical software (www.R-project.org, accessed on 23 November 2021).

## 3. Results

Table 1 displays the descriptive characteristics of the study population by site. Most notably, the NWH population was younger than the MGH and UPenn populations, the UPenn population included nearly 46% Black or African American women, and distributions of age at first birth, breast density, and the proportion of missing data differed across sites. Among the 122,556 women in the study population, 1734 were diagnosed with breast cancer within 5 years of their screening mammogram.

In the full study population (Table 2), the AUCs for the models ranged from 0.590 for BRCAPRO to 0.617 for the BCSC model. We observed significant differences in the AUCs comparing BCRAT and BCSC (*p* = 0.010), BCRAT and BRCAPRO (*p* = 0.007), BCRAT and BRCAPRO+BCRAT (*p* = 0.040), BCSC and BRCAPRO (*p* < 0.001), and BRCAPRO and BRCAPRO+BCRAT (*p* < 0.001). The differences in AUC remained statistically significant after Bonferroni correction for the comparisons of BRCAPRO and BCSC (*p* < 1 × 10^−8^) and BRCAPRO and BRCAPRO+BCRAT. O/E ratios ranged from 1.036 for BCRAT to 1.185 for the BCSC model, and the O/E for the BCSC model was further away from 1. The true positive rate ranged from 30.7% for the BCSC model to 37.8% for the BCRAT model, whereas the false positive rate ranged from 18.4% for BCSC to 24.3% for BRCAPRO. The proportion of patients identified as having high 5-year risk differed across models, with 18.6% of patients identified as high risk based on the BCSC model compared with 24.5% based on the BRCAPRO model. Comparable model performance was observed across the three institutions (Appendix A Table A1). 

**Figure 2:**
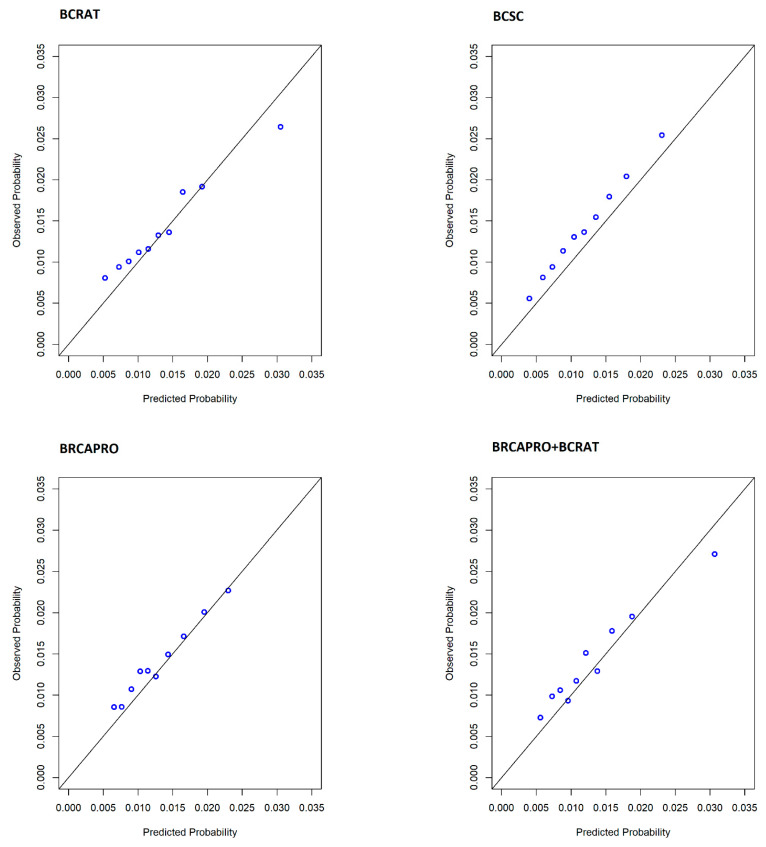
Calibration curves for risk prediction models.

We compared calibration curves for the four models by plotting observed vs. predicted probabilities of breast cancer by deciles (Figure 2). We observed good calibration across deciles for each model. The BCSC model slightly under-predicted the number of cases across all deciles, whereas the BCRAT model under-predicted the number of cases for those in the highest decile. 

Model performance stratified by race/ethnicity is displayed in Table 3. There were 209 breast cancers diagnosed among Black or African American women. Among Black or African American women, the AUCs ranged from 0.610 for BRCAPRO to 0.644 for BCSC, though the differences in AUC by race did not reach statistical significance. In addition, there was no significant difference in AUCs for any model between Black or African American and White women. O/E ratios ranged from 1.054 for BRCAPRO to 1.316 for BCSC. The numbers of cancers among Hispanic (N = 32) and Asian women (N = 54) in the study population were small, so results should be interpreted with caution. In the sensitivity analysis limited to women at UPenn, there was no significant difference in the AUC between Black and White women for the BCRAT or BCRAT+BRCAPRO, but BCSC (*p* = 0.006) and BRCAPRO (*p* = 0.049) had significantly higher AUC among Black compared with White women, though the O/E ratio for both Black (O/E = 1.40) and White (O/E = 1.41) women at UPenn indicated under-prediction of cancer cases for BCSC (Appendix C Table A2). 

Table 4 displays the performance of the risk models by molecular subtype. Given that the models are not trained to predict subtype-specific risk, we did not assess model calibration. AUCs were lower for triple-negative (N = 132, AUC range 0.564–0.585) and HER2+ cancers (N = 224, AUC range 0.513–0.567) compared with ER/PR+HER2− cancers (N = 1316, AUC range 0.605–0.629). The differences in AUCs between ER/PR+HER2− disease and HER2+ disease were statistically significant (all *p*-values < 0.009), but differences did not reach statistical significance for ER/PR+HER2− compared with ER/PR/HER2− (triple-negative breast cancer, TNBC). After Bonferroni correction, the difference in AUC for BRCAPRO between ER/PR+HER2− and HER2+ disease remained statistically significant (*p* < 1 × 10^−5^).

**Figure A1:**
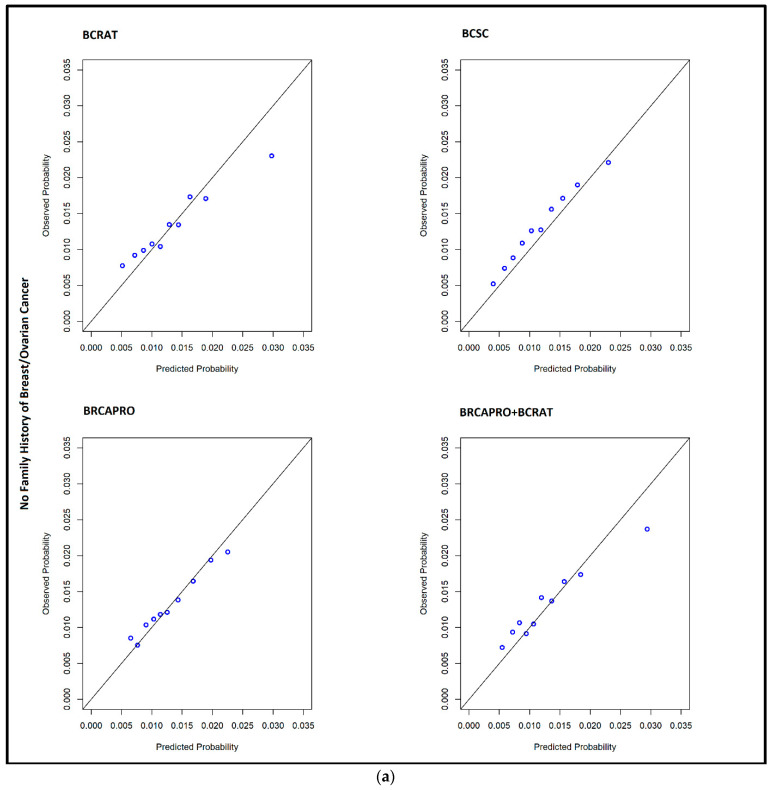

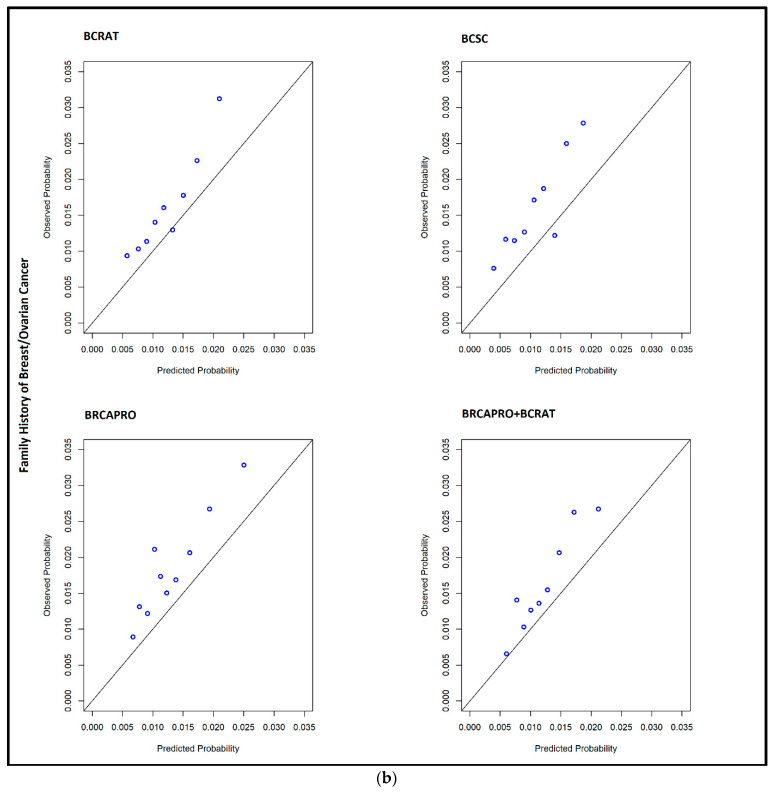
(**a**) Calibration curves for four models for individuals with no family history of breast cancer. (**b**) Calibration curves for four models for individuals with a family history of breast cancer. Each point on the figure represents the observed probability of breast cancer (*Y*-axis) for each decile of probability of breast cancer predicted by the model (*X*-axis). The diagonal line indicates perfect agreement between predicted and observed probability of breast cancer.

Table 5 displays model performance stratified by family history of breast cancer, age, and BMI. For women with a family history, breast cancers were under-predicted for all models (O/E range 1.263–1.509). AUC estimates were significantly higher for patients with family history than without family history for the BCRAT (*p* = 0.019) and BRCAPRO+BCRAT (*p* = 0.016) models. These differences were no longer statistically significant after the Bonferroni correction. O/E ratios for all models among patients with a family history were further from 1 than those without a family history, and confidence intervals did not overlap, suggesting cases were underpredicted for patients with a family history (Appendix B Figure A1). For women under 50, O/E ratios indicated under-prediction of cancers. AUCs did not differ significantly by age. For BMI, all four models had significantly higher AUCs among obese compared with non-obese women (BCRAT *p* = 0.004, BCSC *p* < 0.001, BRCAPRO *p* = 0.001, BRCAPRO+BCRAT *p* = 0.012). Both the BCSC model and the BRCAPRO model had significantly higher AUC among obese women compared with non-obese women after adjusting for multiple comparisons (BCSC *p* = 0.000025, BRCAPRO *p* = 0.0012). However, the under-prediction was more severe for BCSC and BRCAPRO among women with high BMI compared with lower BMI.

Table 6 displays a cross-tabulation of high (≥1.67% 5 years risk) versus low absolute risk classification for all possible pairs of models. Overall, the proportion of discordant classifications ranged from 2.9% for the comparison of BCRAT with BRCAPRO+BCRAT (0.5% + 2.4%) to 19.7% for the comparison of BCSC with BRCAPRO (13.6% + 6.1%). BCSC classified more patients as low risk than other models. Nearly 10% of patients were classified as high risk with the BCRAT model but low risk with BCSC, whereas only 5.7% of patients were classified as low risk on BCRAT and high risk on BCSC. Almost 14% of patients classified as high risk by BRCAPRO were classified as low risk by BCSC, while only 6.1% of patients considered a low risk on BRCAPRO were considered high risk with BCSC. Comparing BCRAT and BRCAPRO, 7.8% of women who were high risk on BCRAT were classified as low risk by BRCAPRO, while 11.1% of women who were high risk on BRCAPRO were classified as low risk by BCRAT. The proportions of patients classified differently by the BRCAPRO+BCRAT model compared with the BCRAT model were smaller.

## 4. Discussion

Our study is one of the largest to examine the validity of multiple breast cancer risk prediction models simultaneously in a large and diverse sample of women undergoing mammography screening, allowing comparisons of model performance by race/ethnicity, tumor molecular subtypes, family history, and obesity. We found that model performance was comparable across the four models examined, with moderate discriminatory accuracy, and generally good calibration, consistent with previous estimates [7,17,18,19,20,21,22,23,24,25,26,27,28,29,30]. The BCSC model had the highest AUC but under-predicted the number of cancer cases to a greater degree than the other models. We found no evidence of poorer model performance for Black or African American women compared with White women. AUC estimates were consistently higher for Black or African American women compared to White women, though not statistically significantly different, except among women at UPenn, where the AUC was better for BCSC and BRCAPRO models for Black compared with White women. With the exception of the BRCAPRO model, the O/E ratio was further from 1 for Black or African American women compared to White women, indicating that models under-predicted the number of cases among Black or African American women. Model discriminatory accuracy was poorer for HER2+ than ER/PR+HER2− disease across all models, and also lower for TNBC than ER/PR+HER2− disease, however, the difference was not statistically significant for TNBC. This is the first study to validate the combined BRCAPRO+BCRAT model, which has significantly better discriminatory accuracy than BRCAPRO in this screening population. BCRAT and BRCAPRO+BCRAT had better AUC among women with family history than women without a family history, though O/E ratios were further from 1 for women with family history compared to those without. Across all models, AUCs were higher for obese women than non-obese women, but the calibration was poorer, with under-prediction of cases among obese women. While overall there was good concordance in the patients identified as high and low absolute risk, we found anywhere from 2.4% to 19.7% discordance in pairwise comparisons of the proportions of patients identified as high versus low risk across the models. These findings add to the literature and provide useful performance metrics to consider when selecting breast cancer risk assessment models, and also highlight the complications when applying risk models clinically and the need for improved risk assessment tools.

The measures of calibration and discrimination of the models observed in our study are generally consistent with existing literature [7,17,18,19,20,21,22,23,24,25,26,27,28,29,30]. This suggests that there is no clear winner in terms of which risk model to use for the purpose of directing screening. However, while we found that the models largely agreed on which patients were high risk, there was variation across models. Interestingly, the BCSC model was more likely to classify women as having low risk when compared with the BCRAT model and the BRCAPRO model. While the choice of model may have only marginal differences at the population level, our results highlight that the choice of model may lead to a different assessment of high-risk status for individual women. In the case of supplemental screening, different recommendations would be made if only one model is used compared to if multiple models are used, and an individual is considered high-risk based on any of the applied models. If the goal is to increase the number of women identified as high risk, we might take the latter approach. If the goal is to minimize the use of resources and unnecessary procedures, we may use only one model or set a higher threshold for high-risk status. Answering such questions is key to implementing a risk-based approach to screening.

Our study found no significant difference in model performance between Black or African American and White women. To our knowledge, ours is the first study to compare the performance of multiple breast cancer risk prediction models simultaneously for White and Black women. AUCs tended to be higher for Black or African American than White women, and calibration tended to be poorer for Black or African American than White women, though *p*-values for differences in AUCs were not significant, and confidence intervals for O/E ratios for Black or African American and White women were overlapping. Despite our large population, there were only 209 breast cancers diagnosed among Black or African American women, which may limit our power to detect differences. The original BCRAT model was found to underestimate risk among Black or African American women and was therefore updated to better predict breast cancer risk for Black or African American women based on the results of the Women’s Contraceptive and Reproductive Experiences (CARE) study [5]. However, even after this update, the BCRAT model was still shown to significantly underestimate risk in a study that included 725 cases and 725 controls from the Black Women’s Health Study [31]. Validation of the BCSC model among women undergoing mammography screening showed that risk was under-predicted for non-Hispanic Black women, though the confidence interval of the observed to expected ratio overlapped with that for White women [28]. The AUC of the BCSC model among Black women was not reported in this study. While discrimination and calibration were comparable across models for Black or African American and White women in our study, all risk models identified a smaller proportion of Black or African American than White women as high risk. For example, over 21% of White women were identified as having high 5 years risk based on the BCRAT model, compared with 10% of Black or African American women. This illustrates the fact that using existing risk models to direct supplemental screening will result in fewer Black or African American women than White women qualifying for additional imaging. While historically Black or African American women in the U.S. have had lower breast cancer mortality than White women, breast cancer incidence has increased for Black or African American women over the past 20 years, with incidence rates now very close to those of White women (127.3 vs. 131.6 per 100,000 women) [1]. Future research should closely evaluate the potential effects of using risk models to guide prevention measures on racial disparities in breast cancer.

Our results further suggest that risk models do not identify the risk of triple-negative or HER2+ tumors as well as hormone receptor-positive HER2− tumors. Our results are consistent with an analysis of the Women’s Health Initiative, which showed that the BCRAT model predicted ER+ breast cancers, but not ER− breast cancers [21] and with our prior analysis with a smaller sample [11]. Given that HER2+ and TNBCs are more aggressive, future studies should attempt to build subtype-specific risk prediction models. Identification of risk of poor prognosis cancers would allow targeting of prevention strategies such as intensive screening to women at greatest risk for breast cancer death. Thus far, the lower prevalence of both HER2+ and triple-negative tumors have limited the ability to generate subtype-specific models.

BRCAPRO and BRCAPRO+BCRAT use more extensive family history data than the BCSC and BCRAT models. In this screening cohort, approximately 20% of patients had any family history of breast cancer, including first or second-degree relatives. We found that BRCAPRO+BCRAT had significantly better AUC than BRCAPRO and calibration was very similar for both models overall, and among the subset of women with family history. This highlights the need to balance between a potentially small increased accuracy of risk assessment including extended family history, and the additional time and computational burden of collecting and analyzing extensive family history data. It is important to note that detailed family history assessment has the added benefit of identifying patients who may be at high risk for high and moderate penetrance mutations such as *BRCA1/2* who may benefit from genetic testing.

We found significantly higher AUC across models among obese women compared with non-obese women, though calibration was poorer for obese women, particularly for the BCSC model. While none of the existing models include BMI as a predictor, the BCSC model does incorporate breast density, which is highly correlated with BMI, with women with higher BMI having lower breast density on average. Therefore, the BCSC model likely downgrades risk for many obese women given their lower breast density, leading to under-prediction of risk. Breast density as coded by the radiologist has been shown to have poor reproducibility [32]. Novel measures of breast density, such as quantitative measures of volumetric breast density from digital breast tomosynthesis may prove a better risk marker than radiologist-coded breast density, particularly for obese women. A recent study found that the effect of volumetric breast density on breast cancer risk was strongest in overweight and obese women [33], suggesting that risk models may need to incorporate these novel measures and include interaction terms between density and BMI in order to improve risk assessment for obese women. Given that the prevalence of obesity is large and increasing, and that obese women are more likely to be diagnosed with more aggressive diseases [34,35], the inclusion of BMI in risk models should be explored, with a closer evaluation of how to best predict risk among women with high BMI.

To our knowledge, this is one of the largest studies to compare multiple breast cancer risk models among women undergoing mammography screening in the U.S. The large and diverse cohort enabled comparisons of model performance by race/ethnicity, molecular subtypes, family history, age, and BMI. Additionally, we were able to evaluate a novel combination of the BRCAPRO and BCRAT model, BRCAPRO+BCRAT, that may be useful for patients with an extensive family history of breast and ovarian cancers. Our findings provide directions for future research by identifying subsets of the population for which existing models may have suboptimal performance.

Several limitations should be noted when interpreting the findings. We were unable to include the IBIS/Tyrer-Cuzick model because we lacked sufficient data on the use of menopausal hormone therapy for input to this model. Due to the small number of Hispanic and Asian/Pacific Islander patients, there were too few cancer outcomes to make meaningful inferences on the performance of the risk models in these racial/ethnic groups. There were 209 breast cancers among Black or African American women, which is also a relatively small sample. Most of the Black or African American patients were screened at one of the three sites, and therefore results for Black or African American patients may be confounded by site-specific measurement biases. In addition, the data on prior biopsy from MGH was incomplete. However, we believe the data on atypical hyperplasia to be reasonably accurate since it incorporated both self-reported data and information extracted from biopsy reports. However, we lacked data on other benign breast conditions, such as non-proliferative benign breast diseases and proliferative benign breast diseases without atypia. While the BCRAT uses atypical hyperplasia, the BCSC model uses benign breast disease, and therefore we may be underestimating risk among patients since we do not have full data on benign breast diseases. This highlights the broader concern, that there is variation in data quality across risk factors and across sites. However, this is the reality when data collected for clinical purposes is utilized for risk assessment. The inclusion of three cohorts with slightly different risk collection instruments may help in reducing the effect of misclassification of risk factors at any one site on the results. We treated patients diagnosed with DCIS as non-cases since the risk models considered focus on the risk of invasive breast cancer. However, since risk factors for DCIS and invasive cancer share common risk factors, this may have led to the poorer observed performance of our models. Additionally, the purpose of this study was to evaluate risk models that would be feasible to integrate into clinical care in mammography to support decision-making in the general population. We excluded known *BRCA1/2* carriers because the BCRAT and BCSC risk models are specifically designed to predict risk in non-carriers. There were fewer *BRCA1/2* carriers at NWH than MGH and UPenn, which is partly expected, as NWH is a community hospital, whereas MGH and UPenn have large cancer genetics clinics. It is also possible that *BRCA1/2* mutation status was less well annotated in health records at NWH, and we may have included some *BRCA1/2* mutation carriers in the analysis, which may bias results in unknown ways. However, given the small prevalence of *BRCA1/2* mutations in the general population, we expect the magnitude of this bias to be small. Finally, we lacked genetic data to incorporate polygenic risk scores into risk models, which has been shown to improve predictive accuracy. A recent study showed that adding the 313 SNP polygenic risk score to classical breast cancer risk factors improved the AUC from 56% to 64% among women younger than 50 and from 57% to 64% among women aged 50 and older [36]. Polygenic risk scores are not yet integrated into clinical practice to direct supplemental screening, and further studies are needed to direct the implementation of such approaches. Finally, we performed a large number of comparisons, and therefore some of the statistically significant associations may have been the result of chance rather than true underlying differences in performance. We provide Bonferroni corrected *p*-values and found several associations that remained statistically significant after Bonferroni correction.

## 5. Conclusions

In summary, our research provides important data on the performance of breast cancer risk assessment models among women undergoing mammography screening in the U.S. and provides important data on how models perform in different subsets of the population, including Black or African American women and women with a family history of breast cancer, and how well the models predict breast cancer subtypes. We validate the BRCAPRO+BCRAT model which may be beneficial for use among women with a family history of breast and ovarian cancer. These findings are useful to develop risk assessment strategies but ultimately point to the need to further improve risk prediction by incorporating additional risk factors, such as quantitative measures of breast density and genetic markers.

## Figures and Tables

**Table 1:** Risk factors by study site.

Risk Factors	MGH	NWH	UPenn
	N = 58,706	N = 39,189	N = 24,661
	N (%)		
Age, mean +/− SD	52.57 +/− 9.55	51.37 +/− 8.82	55.6 +/− 9.29
Age			
40–44 years	15,723 (26.78)	10,796 (27.55)	3966 (16.08)
45–49 years	9811 (16.71)	8380 (21.38)	3851 (15.62)
50–54 years	9246 (15.75)	6976 (17.80)	4154 (16.84)
55–59 years	8862 (15.10)	5245 (13.38)	4226 (17.14)
60–64 years	7047 (12.00)	3845 (9.81)	3787 (15.36)
65–69 years	4724 (8.05)	2400 (6.12)	2857 (11.59)
70–74 years	3293 (5.61)	1547 (3.95)	1820 (7.38)
Race/Ethnicity			
White	48,072 (81.89)	35,394 (90.32)	11,312 (45.87)
Black or African American	3266 (5.56)	630 (1.61)	11,279 (45.74)
Hispanic	3177 (5.41)	392 (1.00)	560 (2.27)
Asian	3027 (5.16)	1545 (3.94)	920 (3.73)
Other/Unknown	1164 (1.98)	1228 (3.13)	590 (2.39)
Age at menarche			
7 to 11 years	10,216 (17.40)	5895 (15.04)	4653 (18.87)
12 to 13 years	29,764 (50.70)	22,502 (57.42)	13,190 (53.49)
≤14 years	15,529 (26.45)	10,643 (27.16)	4445 (18.02)
Missing	3197 (5.45)	149 (0.38)	2373 (9.62)
Age at first birth			
Nulliparous	15,673 (26.70)	7297 (18.62)	5794 (23.49)
<20 years	5721 (9.75)	1671 (4.26)	4731 (19.18)
20–24 years	11,652 (19.85)	5979 (15.26)	5001 (20.28)
25–29 years	11,349 (19.33)	9923 (25.32)	4671 (18.94)
≤30	12,217 (20.81)	14,124 (36.04)	3895 (15.79)
Missing	2094 (3.57)	195 (0.50)	569 (2.31)
Breast density			
Almost entirely fat	4342 (7.40)	755 (1.93)	2359 (9.57)
Scattered fibroglandular tissue	23,145 (39.43)	9608 (24.52)	12,216 (49.54)
Heterogeneously dense	27,112 (46.18)	22,898 (58.43)	9162 (37.15)
Extremely dense	4107 (7.0)	5928 (15.13)	924 (3.75)
Menopausal			
Pre-or peri-menopausal	24,645 (41.98)	19,235 (49.08)	11,971 (48.54)
Post-menopausal	34061 (58.02)	19,954 (50.92)	12,690 (51.46)
BMI, mean (SD)	27.21 (6.39)	26.2 (5.80)	29.46 (7.40)
Prior Breast Biopsy			
None	57,807 (98.47)	32,788 (83.67)	19,764 (80.14)
One	541 (0.92)	4656 (11.88)	4259 (17.27)
Two or more	358 (0.61)	1745 (4.45)	638 (2.59)
Prior atypical hyperplasia/benign breast findings ^1^	849 (1.45)	204 (0.52)	91 (0.37)
No. of first-degree relatives with breast cancer (%)			
None	51,037 (86.94)	32,666 (83.36)	20,756 (84.17)
One	7093 (12.08)	5865 (14.97)	3500 (14.19)
Two or more	576 (0.98)	658 (1.68)	405 (1.64)
No. of second-degree relatives with breast cancer (%)			
None	51,285 (87.36)	28,052 (71.58)	21,893 (88.78)
One	5818 (9.91)	7764 (19.81)	2169 (8.80)
Two or more	1603 (2.73)	3373 (8.61)	599 (2.43)
No. of first or second degree relatives with ovarian cancer (%)			
None	58,646 (99.90)	39,152 (99.91)	23,644 (95.88)
One	58 (0.10)	7 (0.02)	919 (3.73)
Two or more	2 (0.00)	30 (0.08)	98 (0.40)
5-year invasive cancer subtype, N (%)			
ER/PR+HER2−	619 (57.31)	379 (56.48)	318 (55.99)
ER/PR+HER2+	77 (7.13)	55 (8.20%)	30 (5.28)
ERPR−HER2+	29 (2.69)	15 (2.24)	18 (3.17)
ERPR−HER2−	58 (5.37)	36 (5.37)	38 (6.69)
Invasive cancer, missing subtype	21 (1.94)	27 (4.02)	14 (2.46)

^1^ At MGH, patients reported if they’d ever had benign tissue removed from the breast. At both MGH and NWH, we also included pathology report data on atypical hyperplasia, LCIS, or lobular neoplasia. At UPenn, patients self-reported prior atypical hyperplasia.

**Table 2:** Overall performance of risk models ^1^.

Performance Metric	All Sites (N = 1734 Cases & 120,822 Non-Cases)							
	BCRAT		BCSC		BRCAPRO		BRCAPRO+BCRAT	
	Estimate	95% CI	Estimate	95% CI	Estimate	95% CI	Estimate	95% CI
O/E	1.036	0.989, 1.084	1.185	1.130, 1.239	1.076	1.027, 1.125	1.063	1.014, 1.112
AUC ^1^	0.604	0.590, 0.618	0.617	0.603, 0.630	0.590	0.578, 0.603	0.608	0.594, 0.621
TPR	0.378	0.354, 0.402	0.307	0.284, 0.328	0.358	0.338, 0.378	0.347	0.324, 0.368
FPR	0.238	0.235, 0.240	0.184	0.181, 0.186	0.243	0.241, 0.245	0.217	0.215, 0.219
Patients with high 5-year risk, N (%)	29,396 (23.99)	-	22,751 (18.56)	-	30,016 (24.49)	-	26,830 (21.89)	-

^1^ *p*-values for difference in AUC: BCRAT vs. BCSC *p* = 0.010, BCRAT vs. BRCAPRO *p* = 0.007, BCRAT vs. BRCAPRO+BCRAT *p* = 0.040, BCSC vs. BRCAPRO *p* < 0.001, BCSC vs. BRCAPRO+BCRAT *p* = 0.071, BRCAPRO vs. BRCAPRO+BCRAT *p* < 0.001. Bonferroni corrected *p*-values remain significant for BCSC vs. BRCAPRO and BRCAPRO vs. BRCAPRO+BCRAT.

**Table 3:** Performance of risk models by race/ethnicity ^1^.

Performance Metric by Race/Ethnicity	BCRAT		BCSC		BRCAPRO		BRCAPRO+BCRAT	
White (N = 1411 cases)	Estimate	95% CI	Estimate	95% CI	Estimate	95% CI	Estimate	95% CI
O/E	1.031	0.98, 1.082	1.183	1.124, 1.242	1.100	1.045, 1.154	1.063	1.012, 1.116
AUC	0.601	0.588, 0.613	0.607	0.595, 0.621	0.581	0.567, 0.596	0.604	0.59, 0.616
TPR	0.410	0.387, 0.432	0.337	0.311, 0.361	0.378	0.352, 0.404	0.377	0.351, 0.402
FPR	0.270	0.267, 0.273	0.215	0.212, 0.217	0.269	0.266, 0.272	0.244	0.241, 0.246
Patients with high 5-year risk, N (%)	25,777 (27.2)	-	20,503 (21.63)	-	25,628 (27.04)	-	23,275 (24.56)	-
Black or African American (N = 209 cases)								
O/E	1.100	0.964, 1.24	1.316	1.155, 1.483	1.054	0.924, 1.186	1.141	1.000, 1.287
AUC	0.614	0.581, 0.647	0.644	0.606, 0.675	0.610	0.577, 0.646	0.617	0.585, 0.653
TPR	0.321	0.267, 0.382	0.225	0.171, 0.282	0.378	0.319, 0.445	0.287	0.234, 0.350
FPR	0.175	0.169, 0.181	0.103	0.098, 0.107	0.244	0.237, 0.251	0.169	0.164, 0.175
Patients with high 5-year risk, N (%)	2689 (17.72)	-	1584 (10.44)	-	3738 (24.63)	-	2588 (17.05)	-
Hispanic (N = 32 cases)								
O/E	0.748	0.492, 0.983	1.028	0.68, 1.354	0.840	0.552, 1.106	0.934	0.614, 1.223
AUC	0.583	0.489, 0.699	0.582	0.491, 0.671	0.570	0.495, 0.645	0.567	0.467, 0.668
TPR	0.063	0.000, 0.172	0.000	0.000, 0.000	0.000	0.000, 0.000	0.000	0.000, 0.000
FPR	0.051	0.044, 0.057	0.020	0.016, 0.024	0.006	0.004, 0.008	0.024	0.02, 0.029
Patients with high 5-year risk, N (%)	209 (5.06)	-	81 (1.96)	-	24 (0.58)	-	100 (2.42)	-
Asian (N = 54 cases)								
O/E	1.588	1.172, 1.999	1.157	0.860, 1.458	0.962	0.714, 1.212	1.054	0.781, 1.327
AUC	0.557	0.476, 0.641	0.621	0.555, 0.705	0.617	0.542, 0.703	0.588	0.506, 0.656
TPR	0.000	0.000, 0.000	0.037	0.000, 0.093	0.000	0.000, 0.000	0.037	0.000, 0.093
FPR	0.021	0.018, 0.025	0.017	0.014, 0.021	0.004	0.003, 0.006	0.068	0.062, 0.075
Patients with high 5-year risk, N (%)	116 (2.11)	-	96 (1.75)	-	23 (0.42)	-	373 (6.79)	-

^1^ *p*-values for comparisons of AUCs for risk models by race/ethnicity are not statistically significant.

**Table 4:** Performance of risk models by molecular subtypes.

Performance Metric by Subtype	BCRAT ^1^		BCSC ^2^		BRCAPRO ^3^		BRCAPRO+BCRAT ^4^	
ER/PR+HER2− (N = 1316 cases)	Estimate	95% CI	Estimate	95% CI	Estimate	95% CI	Estimate	95% CI
AUC	0.616	0.603, 0.631	0.629	0.615, 0.645	0.605	0.590, 0.621	0.621	0.606, 0.636
TPR	0.390	0.363, 0.417	0.313	0.290, 0.337	0.384	0.360, 0.410	0.358	0.331, 0.384
FPR	0.238	0.236, 0.240	0.184	0.182, 0.186	0.243	0.241, 0.245	0.217	0.215, 0.219
Patients with high 5-year risk, N (%)	29,253 (23.95)	-	22,630 (18.53)	-	29,902 (24.48)	-	26,699 (21.86)	-
All HER2+ (N = 224 cases)								
AUC	0.560	0.525, 0.600	0.567	0.535, 0.610	0.513	0.479, 0.553	0.561	0.526, 0.599
TPR	0.330	0.270, 0.390	0.281	0.236, 0.338	0.246	0.200, 0.307	0.299	0.248, 0.353
FPR	0.238	0.235, 0.240	0.184	0.182, 0.186	0.243	0.241, 0.246	0.217	0.215, 0.219
Patients with high 5-year risk, N (%)	28,794 (23.80)	-	22,266 (18.40)	-	29,438 (24.33)	-	26,278 (21.72)	-
ER/PR/HER2− (N = 132 cases)								
AUC	0.570	0.516, 0.617	0.585	0.546, 0.630	0.564	0.522, 0.604	0.569	0.513, 0.621
TPR	0.348	0.252, 0.430	0.295	0.215, 0.375	0.295	0.216, 0.369	0.303	0.220, 0.380
FPR	0.238	0.236, 0.240	0.184	0.182, 0.186	0.243	0.241, 0.246	0.217	0.215, 0.219
Patients with high 5-year risk, N (%)	28,786 (23.80)	-	22,257 (18.40)	-	29,435 (24.34)	-	26,268 (21.72)	-

^1^*p*-values for differences in AUCs for BCRAT: ER/PR+HER2− vs. HER2+ *p* = 0.008, ER/PR+HER2− vs. TNBC *p* = 0.084. ^2^ *p*-values for differences in AUCS for BCSC: ER/PR+HER2− vs. HER2+ *p* = 0.002, ER/PR+HER2− vs. TNBC *p* = 0.081. ^3^ *p*-values for differences in AUCS for BRCAPRO: ER/PR+HER2− vs. HER2+ *p* < 0.001, ER/PR+HER2− vs. TNBC *p* = 0.100. ^4^ *p*-values for differences in AUCS for BRCAPRO+BCRAT: ER/PR+HER2− vs. HER2+ *p* = 0.003, ER/PR+HER2− vs. TNBC *p* = 0.063.

**Table 5:** Performance of risk models by family history of breast cancer, age, and BMI.

Family History ^1^	No Family History of Breast Cancer (N = 1340 Cases)							
	BCRAT		BCSC		BRCAPRO		BRCAPRO+BCRAT	
	Estimate	95% CI	Estimate	95% CI	Estimate	95% CI	Estimate	95% CI
O/E	0.982	0.934, 1.045	1.115	1.061, 1.186	1.007	0.959, 1.07	1.016	0.966, 1.081
AUC	0.594	0.582, 0.61	0.612	0.601, 0.625	0.588	0.575, 0.601	0.597	0.584, 0.613
TPR	0.352	0.328, 0.377	0.284	0.262, 0.311	0.361	0.339, 0.385	0.316	0.293, 0.342
FPR	0.231	0.229, 0.235	0.179	0.177, 0.181	0.248	0.245, 0.251	0.208	0.205, 0.211
High 5-year risk, N (%)	23,590 (23.3)	-	18,253 (18.03)	-	25,270 (24.96)	-	21,173 (20.92)	-
	Family History of Breast Cancer (N = 394 cases)							
O/E	1.273	1.161, 1.42	1.509	1.38, 1.686	1.402	1.283, 1.569	1.263	1.153, 1.408
AUC	0.633	0.607, 0.659	0.631	0.603, 0.657	0.597	0.571, 0.625	0.636	0.611, 0.666
TPR	0.467	0.42, 0.519	0.386	0.337, 0.428	0.345	0.295, 0.392	0.454	0.405, 0.501
FPR	0.269	0.262, 0.275	0.208	0.202, 0.213	0.220	0.213, 0.227	0.262	0.256, 0.268
High 5-year risk, N (%)	5806 (27.22)	-	4498 (21.09)	-	4746 (22.25)	-	5657 (26.53)	-
Age ^2^	<50 (N = 567 cases)							
O/E	1.150	1.067, 1.251	1.386	1.287, 1.508	1.219	1.134, 1.325	1.144	1.062, 1.244
AUC	0.590	0.568, 0.616	0.617	0.593, 0.642	0.566	0.544, 0.594	0.592	0.569, 0.62
TPR	0.136	0.11, 0.161	0.067	0.048, 0.088	0.019	0.009, 0.03	0.129	0.102, 0.153
FPR	0.055	0.053, 0.057	0.023	0.021, 0.024	0.009	0.008, 0.01	0.052	0.051, 0.054
High 5-year risk, N (%)	2947 (5.61)	-	1216 (2.31)	-	463 (0.88)	-	2788 (5.31)	-
	≥50 (N = 1167 cases)							
O/E	0.989	0.929, 1.034	1.107	1.041, 1.16	1.018	0.958, 1.064	1.028	0.966, 1.075
AUC	0.582	0.564, 0.598	0.595	0.579, 0.608	0.571	0.555, 0.586	0.588	0.57, 0.603
TPR	0.496	0.467, 0.524	0.424	0.396, 0.449	0.522	0.496, 0.549	0.453	0.424, 0.479
FPR	0.376	0.372, 0.379	0.306	0.302, 0.309	0.420	0.417, 0.424	0.341	0.338, 0.345
High 5-year risk, N (%)	26,449 (37.77)	-	21,535 (30.75)	-	29,553 (42.20)	-	24,042 (34.33)	-
Body Mass Index ^3^	BMI < 30 kg/m^2^ (N = 999 cases)							
O/E	1.080	1.019, 1.15	1.188	1.122, 1.265	1.128	1.066, 1.2	1.102	1.040, 1.173
AUC	0.588	0.569, 0.604	0.597	0.58, 0.612	0.568	0.552, 0.586	0.594	0.576, 0.613
TPR	0.353	0.328, 0.382	0.305	0.277, 0.334	0.309	0.289, 0.339	0.327	0.300, 0.358
FPR	0.236	0.233, 0.239	0.205	0.202, 0.209	0.233	0.23, 0.237	0.217	0.213, 0.220
High 5-year risk, N (%)	16,254 (23.76)	-	14,157 (20.7)	-	16,048 (23.46)	-	14,934 (21.83)	-
	BMI ≥ 30 kg/m^2^ (N = 428 cases)							
O/E	1.164	1.054, 1.264	1.525	1.384, 1.661	1.179	1.071, 1.285	1.206	1.091, 1.309
AUC	0.634	0.612, 0.657	0.661	0.639, 0.683	0.617	0.592, 0.639	0.634	0.609, 0.659
TPR	0.428	0.388, 0.468	0.250	0.215, 0.283	0.432	0.389, 0.477	0.390	0.348, 0.435
FPR	0.231	0.226, 0.236	0.111	0.107, 0.114	0.267	0.26, 0.273	0.212	0.208, 0.218
High 5-year risk, N (%)	6268 (23.42)	-	3032 (11.33)	-	7212 (26.94)	-	5755 (21.5)	-

^1^*p*-value for difference in AUCs for women with and without family history BCRAT *p* = 0.019, BRCAPRO+BCRAT *p* = 0.016. No significant associations after Bonferroni correction. ^2^ No statistically significant differences in AUCs for risk models by age. ^3^ *p*-value for difference in AUCs between women with BMI < 30 kg/m^2^ and women with BMI ≥ 30 kg/m^2^: BCRAT *p* = 0.004, BCSC *p* < 0.001, BRCAPRO *p* = 0.001, BRCAPRO+BCRAT = 0.012. Differences for BCSC and BRCAPRO remained statistically significant after Bonferroni correction.

**Table 6:** Concordance between 5-year high-risk estimates across models.

Risk Model	Risk Level	BCSC		BRCAPRO		BRCAPRO+BCRAT	
		N (% of Total)		N (% of Total)		N (% of Total)	
		High	Low	High	Low	High	Low
BCRAT	High	15,995 (13.05)	13,401 (10.93)	17,880 (14.59)	11,516 (9.4)	25,826 (21.07)	3570 (2.91)
	Low	6756 (5.51)	86,404 (70.5)	12,136 (9.9)	81,024 (66.11)	1004 (0.82)	92,156 (75.2)
BCSC	High	-	-	14,297 (11.67)	8454 (6.9)	15,013 (12.25)	7738 (6.31)
	Low	-	-	15,719 (12.83)	84,086 (68.61)	11,817 (9.64)	87,988 (71.79)
BRCAPRO	High	-	-	-	-	17,537 (14.31)	12,479 (10.18)
	Low	-	-	-	-	9293 (7.58)	83,247 (67.93)

## Data Availability

The data underlying this article cannot be shared publicly in order to protect patient privacy. The data may be shared in a de-identified format on reasonable request to the corresponding author.

## Appendix A

**Table A1:** Performance of risk models by site.

Performance Metric by Site	BCRAT		BCSC		BRCAPRO		BRCAPRO+BCRAT	
MGH (N = 804 cases)	Estimate	95% CI	Estimate	95% CI	Estimate	95% CI	Estimate	95% CI
O/E	1.060	0.993, 1.131	1.196	1.119, 1.275	1.057	0.988, 1.126	1.095	1.025, 1.166
AUC	0.595 ^a^	0.576, 0.615	0.620 ^c^	0.604, 0.639	0.587 ^d^	0.567, 0.610	0.603 ^b^	0.585, 0.625
TPR	0.331	0.302, 0.362	0.270	0.244, 0.307	0.340	0.311, 0.375	0.294	0.263, 0.325
FPR	0.204	0.201, 0.207	0.162	0.159, 0.166	0.238	0.235, 0.241	0.186	0.183, 0.188
Patients with high 5-year risk, N (%)	12,089 (20.59)	-	9600 (16.35)	-	14,049 (23.93)	-	10,983 (18.71)	-
NWH (N = 512 cases)								
O/E	0.930	0.850, 1.003	1.039	0.950, 1.123	1.026	0.938, 1.107	0.947	0.866, 1.022
AUC	0.618	0.592, 0.640	0.624 ^c^	0.602, 0.646	0.599 ^d^	0.574, 0.622	0.622	0.598, 0.644
TPR	0.408	0.368, 0.452	0.369	0.329, 0.406	0.324	0.281, 0.365	0.387	0.345, 0.433
FPR	0.252	0.248, 0.256	0.218	0.213, 0.222	0.205	0.201, 0.209	0.228	0.224, 0.232
Patients with high 5-year risk, N (%)	9960 (25.42)	-	8603 (21.95)	-	8078 (20.61)	-	9015 (23.00)	-
UPenn (N = 418 cases)								
O/E	1.146	1.044, 1.261	1.402	1.275, 1.544	1.186	1.080, 1.306	1.174	1.070, 1.294
AUC	0.598 ^e^	0.567, 0.625	0.603 ^c^	0.578, 0.631	0.576 ^d^	0.549, 0.603	0.597	0.568, 0.624
TPR	0.433	0.380, 0.477	0.304	0.265, 0.344	0.433	0.389, 0.475	0.402	0.356, 0.447
FPR	0.296	0.291, 0.301	0.182	0.177, 0.187	0.318	0.313, 0.323	0.275	0.270, 0.280
Patients with high 5-year risk, N (%)	7347 (29.79)	-	4548 (18.44)	-	7889 (31.99)	-	6832 (27.70)	-

^a^ *p* < 0.05 for Gail AUC vs. BCSC within same site. ^b^ *p* < 0.05 for Gail AUC vs. BRCAGAIL AUC within same site. ^c^ *p* < 0.05 for BCSC AUC vs. BRCAPRO AUC within same site. ^d^ *p* < 0.05 for BRCAPRO AUC vs. BRCAGAIL AUC within same site. ^e^ *p* < 0.05 for Gail AUC vs. BRCAPRO AUC within same site.

## Appendix C

**Table A2:** Performance of risk models by race/ethnicity at UPenn.

Performance Metric by Race	BCRAT		BCSC		BRCAPRO		BRCAPRO+BCRAT	
White (N = 220 Cases)	Estimate	95% CI	Estimate	95% CI	Estimate	95% CI	Estimate	95% CI
O/E	1.129	0.999, 1.281	1.400	1.241, 1.588	1.259	1.117, 1.434	1.161	1.027, 1.317
AUC	0.580	0.544, 0.619	0.565 ^a^	0.525, 0.601	0.549 ^b,c^	0.513, 0.583	0.583	0.548, 0.623
TPR	0.532	0.47, 0.595	0.373	0.309, 0.431	0.473	0.402, 0.533	0.500	0.436, 0.561
FPR	0.421	0.412, 0.429	0.273	0.264, 0.281	0.401	0.394, 0.41	0.383	0.373, 0.391
Patients with high 5-year risk, N (%)	4783 (42.28)	-	3112 (27.51)	-	4557 (40.28)	-	4354 (38.49)	-
Black or African American (N = 170 cases) ^d^								
O/E	1.152	0.984, 1.377	1.413	1.218, 1.691	1.112	0.957, 1.33	1.190	1.016, 1.423
AUC	0.610	0.571, 0.653	0.644	0.594, 0.684	0.608	0.565, 0.648	0.615	0.572, 0.659
TPR	0.353	0.276, 0.424	0.241	0.178, 0.305	0.429	0.353, 0.5	0.324	0.263, 0.393
FPR	0.199	0.192, 0.206	0.110	0.105, 0.116	0.273	0.265, 0.282	0.193	0.186, 0.2
Patients with high 5-year risk, N (%)	2266 (20.09)	-	1267 (11.23)	-	3108 (27.56)	-	2201 (19.51)	-

^a^ *p* < 0.05 between White vs. Black women for BCSC model. ^b^ *p* < 0.05 between White vs. Black women for BRCAPRO model. ^c^ *p* < 0.05 between BRCAPRO vs. BRCAPRO+BRAT models for White women. ^d^ *p* < 0.05 between BRCAT vs. BCSC, BCSC vs. BCRAPRO, & BCSC vs. BRCAPRO+BRCAT for Black women.
